# Dietary and nutritional interventions in the treatment of childhood neuropsychiatric disorders: evidence and myths

**DOI:** 10.1016/j.jped.2025.101465

**Published:** 2025-10-30

**Authors:** Regina Torres Duarte Kostenko, Nathalia Ferreira Antunes de Almeida, Juliana Fernandez Santana e Meneses

**Affiliations:** a Universidade Federal de São de São Paulo (UNIFESP), Departamento de Pediatria, São Paulo, SP, Brazil; b Universidade Federal do Estado do Rio de Janeiro, Escola de Nutrição, Departamento de Nutrição Aplicada, Rio de Janeiro, RJ, Brazil

**Keywords:** Neurodevelopmental disorders, Autism spectrum disorder, Child nutrition sciences, Nutritional supplementation, Attention deficit disorder with hyperactivity

## Abstract

**Objective:**

To analyze the current scientific literature on the main dietary and nutritional interventions proposed for children and adolescents with neuropsychiatric disorders and describe their efficacy and safety, differentiating evidence-based practices from common myths.

**Data source:**

The search was conducted using PubMed, SciELO, Cochrane, and Web of Science databases (2019–2025). The specific terms used in the search were formulated to encompass dietary interventions in children and adolescents with neuropsychiatric disorders and their outcomes.

**Data synthesis:**

Gluten-free and casein-free (GFCF) diets did not reduce ASD symptoms and should be reserved for confirmed allergy or intolerance. Probiotics have shown inconsistent results for core ASD/ADHD symptoms, although they may alleviate gastrointestinal complaints in subgroups. Omega-3 s have shown modest and heterogeneous effects; In ADHD, high-dose EPA may improve attention when baseline levels are low, without consistent benefit in other domains. N-acetylcysteine reduced irritability in some trials, with no consistent effect on core symptoms. Folinic acid showed benefits in subgroups defined by biomarkers (folate receptor autoantibodies) and in ASD with language impairment, but this needs to be confirmed in larger studies. For vitamin D, high rates of insufficiency and possible modest gains in sub-outcomes were observed, with methodological heterogeneity.

**Conclusions:**

This literature review showed that there is no scientific support for generalized dietary interventions, such as restrictive diets or nutritional supplementation, in the treatment of childhood neuropsychiatric disorders. Current evidence suggests the need for individualization and continuous monitoring. Specific interventions are justified only in the case of documented nutritional deficiencies.

## Introduction

Neurodevelopmental disorders constitute a group of heterogeneous conditions, beginning early in childhood development, that lead to functional impairments and impact the quality of life of children and their families in the short and long term. This group includes: Autism Spectrum Disorder (ASD), Attention Deficit/Hyperactivity Disorder (ADHD), Motor Neurodevelopmental Disorders, Anxiety Disorders, among others [1].

In addition to general cognitive and behavioral symptoms, the involvement of the microbiota in gastrointestinal disorders that coexist with neuropsychiatric conditions has been investigated. This complex link between the gastrointestinal tract and the central nervous system (CNS) is termed the "gut-brain axis" and involves neural, immunological, neuroendocrine, and metabolic pathways [2].

In this context, interest in dietary and nutritional interventions has grown as a promising strategy for improving symptoms, both gastrointestinal and CNS-related. The main hypothesis is that nutrition plays an important role in modulating the gut-brain axis and regulating inflammatory processes, which may be associated with the pathophysiology of various neuropsychiatric disorders, including alterations in lipid metabolism and neuroinflammation [3, 4, 5]. Several factors can alter the diversity of the gut microbiota, such as infections, antibiotic exposure, type of delivery (vaginal or cesarean section), dietary patterns, breastfeeding, psychophysiological stress, and genetic factors. Furthermore, maternal conditions (infection, obesity), low birth weight, prematurity, and antibiotic use in childhood also modulate its composition [2]. Among the most popular nutritional approaches are exclusion diets, such as the gluten-free and casein-free diet, based on the hypothesis that certain dietary proteins could trigger immune or inflammatory responses that affect the brain [6]. Other explored strategies include supplementation with omega-3 fatty acids, which are essential for neuronal structure and function [7]. As well as the use of vitamins, minerals, and probiotics, aiming to restore the balance of the intestinal microbiota [5]. Despite the growing interest in the topic, the studies published to date present diverse methods and controversial results [6,7]. There is still a lack of randomized controlled clinical trials with adequate sample sizes and long-term follow-up, which hinders evidence-based recommendations for the indiscriminate use of these nutritional interventions [6,7]. Furthermore, exclusion diets can expose children to the risk of nutritional deficiencies and ineffective treatments, which is why their adoption must be judicious and individualized [6].

Given this scenario, the objective of this literature review was to carefully analyze the current evidence related to the main dietary and nutritional interventions used in children and adolescents with neuropsychiatric disorders and to describe the efficacy and safety of these approaches, distinguishing evidence-based findings from common myths.

## Method

A narrative review with a structured search was conducted to map evidence on the efficacy and safety of dietary and nutritional interventions in children and adolescents (0–18 years) with neuropsychiatric disorders, including Autism Spectrum Disorder (ASD), Attention-Deficit/Hyperactivity Disorder (ADHD), anxiety disorders, and depressive disorders.

The research question was organized using the PICO criterion: pediatric population with the aforementioned disorders; dietary and nutritional interventions (such as exclusion diets; eating patterns; probiotics; omega-3 fatty acids; vitamin D; folinic acid; N-acetylcysteine; and micronutrient supplementation); comparison (placebo, usual care, or control intervention); and outcomes: improvement in symptoms related to the central nervous system (CNS) or gastrointestinal tract.

Searches were conducted in the PubMed, SciELO, Cochrane, and Web of Science databases from April to August 2025, combining MeSH and free-text terms with Boolean operators for conditions (autism, ADHD, anxiety, depression), populations (infant, child, adolescent), interventions (diet therapy, nutrition therapy, gluten-free, casein-free, probiotics, omega-3, folinic/leucovorin, vitamin D, N-acetylcysteine, dietary supplements), and outcomes (treatment outcome, therapeutics, safety, and effectiveness).

Filters were applied for humans, age group 0–18 years, randomized controlled trials (RCTs), and systematic reviews/meta-analyses, prioritizing publications from 2019 to 2025; studies prior to this period were included when clinically relevant (e.g., RCTs or reference meta-analyses). Studies in humans diagnosed with neuropsychiatric disorders that evaluated dietary/nutritional interventions and reported efficacy and/or safety outcomes were included. These included clinical trials (randomized and non-randomized), observational studies (prospective/retrospective cohorts and case-control studies), and systematic reviews (with or without meta-analysis). Animal studies, letters to the editor, conference abstracts, case reports, and articles without outcomes of interest were excluded.

Title and abstract screening was followed by full-text reading and data extraction on population, intervention, comparison, and outcomes.

Qualitative assessment considered sample size, blinding/randomization, protocol heterogeneity, risk of bias, and conflicts of interest.

## Results

Among the studies included in this review, RCTs investigating N-acetylcysteine (NAC) and folinic acid in children with ASD, as well as omega-3 supplementation in ADHD, were identified and analyzed. The authors found a limited number of studies on micronutrient supplementation. Observational studies primarily addressed the relationship between nutritional status and neuropsychiatric outcomes, without analyzing the therapeutic efficacy of interventions. Systematic reviews with meta-analyses on the use of probiotics found no evidence for improvement in core symptoms.

### Probiotic use and the gut-brain axis

The bidirectional communication of the gut-brain axis involves neural, immunological, neuroendocrine, and metabolic pathways; alterations in the microbiota (dysbiosis) have been described in neurodevelopmental disorders such as ASD and ADHD [8]. From a mechanistic perspective, dysbiosis can increase intestinal permeability, promote inflammatory processes, and alter the production of neurotransmitters and short-chain fatty acids, with potential repercussions on brain function and behavior [8]. In this sense, modulation of the microbiota by probiotics (live microorganisms) has been proposed as a strategy to alleviate gastrointestinal symptoms and, eventually, neurobehavioral aspects [8].

However, clinical evidence in pediatric populations remains inconsistent. In ASD, a recent meta-analysis of randomized controlled trials demonstrated no benefit from probiotics on core behavioral symptoms, although some studies suggest improvement in gastrointestinal symptoms [9]. In ADHD, a meta-analysis involving 379 children and adolescents found no difference between probiotics and placebo for inattention or hyperactivity/impulsivity [10]. Overall, the results are limited by small sample sizes, strain/dose heterogeneity, short intervention duration, and variation in outcomes and scales [9,10].

More broadly, a systematic review focused on the microbiota-gut-brain axis in psychiatric disorders did not demonstrate a consistent benefit of probiotics/prebiotics for symptoms of stress and anxiety and highlights the need for larger trials, more homogeneous interventions, and clinically relevant outcomes to elucidate their therapeutic potential [8].

Therefore, to date, the hypothesis that probiotics reduce core symptoms of ASD and/or ADHD is not supported by robust evidence, and their routine use is not recommended, although gastrointestinal benefits may occur in specific subgroups [9,10].

### Gluten- and casein-restricted diets

The gluten- and casein-free (GFCF) diet is one of the most widespread, but also one of the most controversial, nutritional interventions for ASD [11,12]. This approach is based on the so-called "opioid theory," according to which peptides derived from gluten and casein, in individuals with increased intestinal permeability, could cross the blood-brain barrier and exacerbate behavioral symptoms [11].

However, recent systematic reviews have found no consistent support for this hypothesis in clinical studies. A meta-analysis, which included six randomized controlled trials, demonstrated no significant benefit of the GFCF diet on autism symptoms, behavior, or functional level [11]. Keller A et al. found similar results in another systematic review and reinforced the low quality and heterogeneity of the available evidence [12].

Clinical guidelines agree and reinforce this conclusion. The National Institute for Health and Care Excellence (NICE) recommends against using exclusion diets, such as GFCF, to treat the core symptoms of autism in children under 19 years of age [13]. The American Academy of Pediatrics (AAP) emphasizes that the evidence for special diets is limited and that interventions should be based on robust data [14]. The European Society for Paediatric Gastroenterology, Hepatology, and Nutrition (ESPGHAN), in its guideline for the diagnosis of celiac disease, establishes that the gluten-free diet is indicated exclusively for patients with confirmed celiac disease and, therefore, does not support the use of a gluten-free diet in ASD in the absence of a specific gastrointestinal condition [15].

Therefore, to date, there is no evidence-based indication for the routine use of the GFCF diet in ASD. Its use should be restricted to cases of confirmed allergy or intolerance to gluten or cow's milk protein, always with nutritional monitoring to prevent deficiencies and prevent developmental impairment.

### Omega-3 fatty acid supplementation

Omega-3 polyunsaturated fatty acids, such as eicosapentaenoic acid (EPA) and docosahexaenoic acid (DHA), are essential components of neuronal membranes. They modulate cell membrane fluidity and neurotransmitter function and exert anti-inflammatory and neuromodulatory properties through the formation of resolvins and other bioactive lipid mediators [16,17]. The hypothesis that omega-3 supplementation benefits children with ADHD has been investigated; however, the results remain controversial.

A double-blind, randomized, controlled clinical trial in children with ADHD compared a high dose of EPA (1.2 g/day) with placebo and found that the EPA-treated group, specifically those with low baseline endogenous levels, showed improvement in focused attention. However, for impulsivity, the improvement in this group was smaller than that observed in the placebo group. Conversely, individuals with higher baseline EPA levels showed less improvement in other ADHD and emotional symptoms. The authors concluded that EPA treatment may be beneficial for cognitive symptoms in individuals with ADHD, only in those with low baseline endogenous levels. Conversely, individuals with high EPA levels may be negatively affected by this treatment [18].

Other clinical trials have shown heterogeneous results and contradictory conclusions. A meta-analysis evaluating clinical trials comparing pharmacological interventions or dietary supplements with placebo in children with ASD demonstrated that the efficacy of dietary supplements was inconclusive. Regarding omega-3 fatty acids, a trend toward improvement in social communication difficulties was observed; however, the effect was limited and based on very low-quality evidence from ten studies in children and adolescents. These studies were limited by the fact that they were clinical trials with small sample sizes and short durations [19].

In summary, although there is biological plausibility for the use of omega-3 in neurodevelopment[16,17] and evidence of limited benefit from EPA in subgroups with low baseline levels in ADHD [18], clinical consistency is still limited, and efficacy in ASD remains inconclusive based on low-quality evidence [19]. Therefore, supplementation should not be routinely recommended and should be considered on a case-by-case basis, preferably guided by nutritional markers (e.g., baseline omega-3/EPA ratio) and integrated into the therapeutic plan. Future research is needed, with appropriate methodological rigor, a larger number of participants, standardized formulations/doses (EPA vs. DHA), and controlled and relevant outcomes for a safe, evidence-based response [16, 17, 18, 19].

### Micronutrients

Vitamin and mineral deficiencies can negatively impact neurocognitive function in children and adolescents. Micronutrient supplementation has been studied as a promising intervention, but its indication should be guided by proven deficiencies, since its indiscriminate use may be ineffective or even harmful [20].

### Zinc

Zinc is a cofactor for numerous enzymes and modulates neurotransmission. Zinc deficiency induces degenerative disorders and cognitive decline, such as increased neuronal death and decreased learning [21]. In children with ADHD, randomized clinical trials suggest that zinc supplementation modestly reduces global symptom scores compared to control/placebo [22]. In children with ADHD, a 12-week RCT showed that zinc monotherapy produced modest benefits, particularly in hyperactivity/impulsivity and socialization, with the effect more evident in subgroups with lower baseline zinc; there was no clear improvement in attention [23].

### Iron

Iron is essential for dopamine synthesis and myelination, fundamental processes in attention and cognition. Systematic reviews and meta-analyses indicate that low ferritin levels are associated with a greater risk and severity of ADHD, and that supplementation may improve symptoms in deficient individuals [24].

### Magnesium

Magnesium is crucial for neuronal excitability and modulation of the stress response. However, evidence on its supplementation in ADHD and ASD remains inconsistent. Recent reviews highlight potential benefits in reducing irritability and anxiety but reinforce the need for robust randomized clinical trials [25].

### Folinic acid

The folate cycle has been described as being related to the pathophysiology of ASD because it plays a role in amino acid and DNA methylation reactions, the glutathione oxidative stress pathway, and the neurotransmitter synthesis pathway [26,27]. Folinic acid, or 5-formyl tetrahydrofolic acid, is the reduced, natural form of folic acid and is clinically known as leucovorin. It is readily converted to the active folate derivative, 5-methyltetrahydrofolate. The l-isomer of folinic acid (levofolinic acid) is the functional form of the isomers; the d-isomer is inactive in the folate metabolic pathway, but high levels of it can interfere with the pathway [27].

The literature recognizes that folate transport across the choroid plexus epithelium occurs via a folate transporter alpha (FRα) through an energy-dependent endocytosis mechanism [28]. Folate deficiency in the cerebrospinal fluid (CSF), termed cerebral folate deficiency (CFD), with normal serum levels, was first documented in six children with neurodevelopmental regression and neurological abnormalities. Thus, treatment with the reduced form of folic acid (called folinic acid) was able to normalize CSF folate concentrations, with consequent improvement in neurological symptoms [29]. Cerebral folate deficiency (CFD) has been reported in the literature as one of the conditions present in children with neurological disorders. The clinical characteristics of CFD manifest from 4–6 months of age and extend over the next two years, until the full clinical phenotype appears. Around 4 to 6 months of age, the child presents the first signs, such as agitation, restlessness, and insomnia, which are followed by slowed brain growth, psychomotor retardation with hypotonia and ataxia, distal pyramidal signs, and, in one-third of patients, dyskinesias and/or epileptic seizures occur. If CFD is left untreated, bilateral visual and hearing loss may occur at a later stage [30].

Some studies suggest that oral treatment with folinic acid leads to improvement in neurological symptoms such as epilepsy, irritability, cognitive decline, ataxia, among others [31,32].

The double-blind, randomized, placebo-controlled study by Frye et al. proposed supplementation with 2 mg/kg/day with a maximum of 50 mg/day for 12 weeks versus placebo for 48 children diagnosed with ASD and language impairment, also separated by subtypes of glutathione-folate receptor alpha autoantibody status. It was found that treatment with high-dose folinic acid resulted in improved verbal communication compared to placebo, especially in the autoantibody-positive subtypes [33].

Panda et al., in a randomized, double-blind, placebo-controlled clinical trial, evaluated a sample of 40 children (2 to 10 years old) in each group, all diagnosed with ASD, with folinic acid supplementation of 2 mg/kg/day with a maximum of 50 mg/day for 24 weeks. Oral folinic acid supplementation was effective and safe in improving the behavioral symptoms of ASD, with more pronounced benefits in children with high autoantibody titers [31].

Ramaekers et al. evaluated 25 children with low-grade ASD and neurological abnormalities. Reduced CSF folate was identified in 92 % of the sample, and in 82 % of them, this reduction was associated with the presence of folate receptor autoantibodies. Supplementation with folinic acid (1.0 mg/kg/day) was able to restore CSF folate status after 12 months and reduce symptoms such as restlessness, irritability, and insomnia in 88 % of the sample [30].

A randomized, double-blind, placebo-controlled clinical trial evaluated the effects of folinic acid therapy (2 mg/kg to 50 mg/day for 10 weeks) in combination with risperidone (Risperdal) in children aged 4 to 12 years diagnosed with ASD. Supplementation resulted in significant improvement in inappropriate speech and other behavioral symptoms of ASD. Significant improvements were observed over time and with treatment interaction in inappropriate speech, stereotypic behavior, and hyperactivity/nonconformity subscale scores. Conversely, no significant improvements were found in lethargy/social withdrawal and irritability [34].

### Vitamin D

The relationship between vitamin D and children's mental health has received increasing attention in recent years. Vitamin D deficiency is highly prevalent in pediatric populations with psychiatric diagnoses, including ASD, ADHD, and internalizing disorders. In a cross-sectional study involving 93 children and adolescents with psychiatric diagnoses, Muskens et al. observed that approximately 77 % had vitamin D deficiency, with this condition being more common in boys with ASD and in patients with a higher body mass index [35]. This finding reinforces the hypothesis that lifestyle factors and clinical predisposition may contribute to both vitamin deficiency and neuropsychiatric vulnerability.

From a clinical perspective, randomized trials evaluated in recent meta-analyses suggest that vitamin D supplementation may mitigate some symptoms in children with ASD. Zhang et al. synthesized data from randomized trials and found significant improvements in symptoms of stereotypy, although they did not observe consistent effects on the global core symptoms of the disorder [36]. Additionally, Song et al. reported that supplementation resulted in significant reductions in scores on standardized scales, such as the Social Responsiveness Scale (SRS) and the Childhood Autism Rating Scale (CARS), suggesting benefits in social and behavioral aspects [37]. Despite the positive signs, both meta-analyses highlight recurring limitations: small sample size, short follow-up duration, and heterogeneity in doses and protocols used.

In addition to the direct clinical effects, there is also a primary prevention perspective. A Danish cohort study involving nearly 72,000 newborns demonstrated that low vitamin D levels in the neonatal period were associated with an increased future risk of schizophrenia, ADHD, and ASD [38]. This population-based evidence strengthens the idea that vitamin D plays a modulatory role in neurodevelopment and that early deficiency may constitute a shared risk factor among different neuropsychiatric disorders.

Therefore, although the literature still presents heterogeneous results, the most robust body of evidence suggests that vitamin D is involved in multiple aspects of childhood mental health: from the increased prevalence of deficiency in clinical populations, to possible symptomatic benefits in ASD when used as adjunctive supplementation, to the association between low neonatal levels and a higher risk of developing psychiatric disorders in adulthood. However, multicenter studies with larger numbers of participants and standardized protocols remain necessary to confirm therapeutic efficacy and establish consistent clinical recommendations.

Finally, micronutrient supplementation in ADHD and ASD should be strictly guided by clinical and laboratory evaluation, prioritizing the correction of confirmed deficiencies. Indiscriminate supplementation is not recommended.

### The role of the anti-inflammatory diet and the harms of ultra-processed foods

The relationship between diet and neurodevelopment has received increasing attention, particularly due to the impact of dietary patterns on systemic inflammation and oxidative stress—mechanisms implicated in conditions such as ASD and ADHD. Recent evidence suggests that adopting an anti-inflammatory diet and reducing the consumption of ultra-processed foods may act as complementary, low-risk strategies [39].

The anti-inflammatory diet is characterized by a high intake of fruits, vegetables, nuts, seeds, omega-3-rich fish, olive oil, and whole grains—foods with antioxidant and anti-inflammatory properties [39,40]. These dietary patterns modulate the immune response and the microbiota-brain axis, potentially reducing chronic low-grade inflammation and protecting the integrity of barriers (such as the blood-brain barrier) and neuronal signaling, which aligns with a better mental health profile when compared to patterns rich in ultra-processed foods [40].

In contrast, ultra-processed foods—rich in added sugars, saturated fats, sodium, and artificial additives—are associated with the induction and perpetuation of systemic inflammation [41]. Observational studies and meta-analyses indicate that higher consumption of these foods is linked to poorer mental health and increased ADHD symptoms [41]. Furthermore, ultra-processed foods negatively affect the gut microbiota, favoring dysbiosis and dysfunction of the gut-brain axis [42].

Another notable mechanism is the formation of advanced glycation end products (AGEs), abundant in ultra-processed diets. These compounds cross the blood-brain barrier, promote oxidative stress, and activate the AGE–AGE receptor axis, which has been linked to brain aging and neuropsychiatric disorders [43,44].

Thus, diets low in essential fatty acids and high in ultra-processed foods can intensify oxidative and inflammatory pathways, reducing the availability of fundamental structural lipids to the brain. Although the findings are still preliminary, evidence suggests that anti-inflammatory dietary patterns represent a biologically plausible and safe strategy as an adjunctive intervention in neurodevelopmental disorders.

### N-acetylcysteine (NAC)

N-acetylcysteine (NAC) is a derivative of l-cysteine, recognized for its ability to increase glutathione levels and modulate glutamatergic neurotransmission. These mechanisms support their interest as an adjunctive intervention in neurodevelopmental disorders, especially in ASD, in which oxidative stress and excitotoxicity play a relevant role in the pathophysiology [45].

Neuronal dysfunction, caused by elevated exposure to reactive oxygen species, is identified as one of the mechanisms involved in psychiatric and neurodevelopmental conditions, although evidence is still limited [46]. In this context, clinical studies in pediatric populations have sought to evaluate the efficacy of NAC, especially in ASD.

Accumulating clinical evidence suggests that NAC may have positive effects on specific behavioral symptoms. One of the first randomized clinical trials, conducted by Hardan et al., demonstrated that NAC in doses up to 2700 mg/day significantly reduced irritability in children with ASD, as measured by the ABC-I subscale [46]. In contrast, a subsequent, larger, and longer-term study (12 weeks) conducted by Wink et al. found no significant improvement on social and communication symptom scales, although it confirmed an increase in systemic glutathione levels [47].

A consolidated meta-analysis published in the Australian and New Zealand Journal of Psychiatry, gathering data from randomized clinical trials, concluded that NAC significantly reduces irritability and hyperactivity, as well as improves social perception, while maintaining a good tolerability profile [48]. More recently, a review published in 2024 highlighted NAC as one of the most promising adjunctive interventions in ASD, but emphasized that the evidence is still heterogeneous and insufficient for definitive clinical recommendations [49].

Thus, although NAC has a favorable safety profile and consistent results on symptoms such as irritability and repetitive behaviors, the findings are still limited by small sample sizes, short follow-up duration, and methodological variability. Larger, long-term multicenter trials are needed to confirm its efficacy and better define its role in the clinical management of children with ASD.

## Discussion

The body of evidence on dietary and nutritional interventions for childhood neuropsychiatric disorders remains heterogeneous. For omega-3, RCTs and syntheses indicate modest and uneven effects, with possible moderation by baseline status (e.g., low EPA), composition (EPA vs. DHA), dose, and duration. For N-acetylcysteine, there are signals in specific behavioral domains (such as irritability), but the lack of a consistent effect on core symptoms and methodological variability limit conclusions. Folinic acid showed benefit mainly in biologically defined subgroups, such as children positive for folate receptor autoantibodies. This suggests that phenotyping and biomarkers can determine the response; however, larger, standardized RCTs are lacking. For vitamin D, the high prevalence of insufficiency in clinical samples and some positive secondary outcomes contrast with inconsistent results across studies. For GFCF diets and probiotics, the meta-analyses do not support improvement in core symptoms, although gastrointestinal symptoms may improve in subgroups.

An evaluation of the most popular nutritional interventions reveals a contrasting scenario, in which promises of benefit coexist with a lack of robust data for other approaches. The challenge lies in translating heterogeneous evidence into clinical practice safely and ethically. Healthcare professionals must act as knowledge mediators, conducting thorough and individualized nutritional assessments and communicating transparently the difference between evidence and myth, in addition to warning about the risks of unsupervised restrictive diets, excessive consumption of ultra-processed foods, and the indiscriminate use of supplements.

As a basis for care, a diet based on the Dietary Guidelines for the Brazilian Population and the Dietary Guidelines for Brazilian Children Under 2 Years of Age is recommended — prioritizing natural and minimally processed foods and restricting ultra-processed foods [50,51].

In general, recurring limitations such as small sample sizes, short duration, heterogeneous outcomes (often not focused on clinically significant outcomes), varied protocols and formulations, risk of bias, and lack of stratification by biomarkers/phenotypes reduce the precision and generalizability of effect estimates. Furthermore, the isolated effect of nutrition is difficult to measure in multifactorial conditions, in which comorbidities, behavioral interventions, and pharmacotherapy interact. The literature suggests that subgroups (defined by nutritional status, immune/metabolic markers, and clinical profiles) may respond differently, highlighting the need for clinical trials with functional and quality-of-life outcomes and sufficient follow-up to assess sustained changes.

## Conclusion

The lack of scientific support prevents the recommendation of a rigid dietary model for pediatric neurological disorders, nor the supplementation of specific nutrients.

Specific nutritional interventions, when justified by evidence and clinical evaluation (ideally supported by biomarkers), should be carefully integrated and monitored.

Nutrition is a vital pillar for child health and development, and adequate and healthy nutrition should always be the essential foundation of any therapeutic plan. Specific nutritional interventions, when justified by evidence and clinical evaluation, should be carefully and safely integrated. The best care for children with neuropsychiatric disorders consists of a comprehensive, evidence-based, and multidisciplinary approach, combining knowledge from different areas of healthcare to optimize each child's potential.

## Sources of funding

Not applicable.

## Data availability

The data that support the findings of this study are available from the corresponding author.

## Conflicts of interest

The authors declare no conflicts of interest.
